# Native T1 and T2 values by Cardiovascular Magnetic Resonance Imaging in patients with systemic inflammatory conditions

**DOI:** 10.1186/1532-429X-16-S1-P251

**Published:** 2014-01-16

**Authors:** Rocio Hinojar, Lucy Foote, Eduardo Arroyo Ucar, Ning Binti Ngah, Nancy Kuo, David D'Cruz, Bernhard Schnackenburg, David M Higgins, Tobias Schaeffter, Eike Nagel, Valentina Puntmann

**Affiliations:** 1 Cardiovascular Imaging Department, King's College London, London, UK; 2 Philips Healthcare, London, UK

## Background

Patients with systemic inflammatory diseases are at risk of heart failure due to sustained systemic inflammation leading to diffuse myocardial injury and left ventricular remodelling. Because native T1 and T2 values are raised in the presence of diffuse fibrosis and oedema, T1 and T2 mapping by cardiovascular magnetic resonance (CMR) are emerging as potential tools to assess diffuse myocardial involvement. In this study, we examined native T1 and T2 values in patients with systemic inflammatory diseases.

## Methods

79 patients with a clinical diagnosis of systemic inflammatory disease (systemic lupus erythematosus (SLE, n = 46), rheumatoid arthritis (RA, n = 17), systemic sclerosis (SS, n = 9) and Wegener's granulomatosis (WG, n = 7) underwent CMR study for routine assessment of oedema, function and scar at 3-Tesla. 36 healthy subjects served as controls. Native T1 values were acquired using 3'3'5 MOLLI and measured conservatively within septal myocardium of midventricular short-axis slice (mSAX). T2 values were recorded from T2 maps based on GraSE sequence using equivalent cardiac geometry. We compared regional T2 values between patients and controls, and assessed associations with native T1 values.

## Results

**Figure 1:**
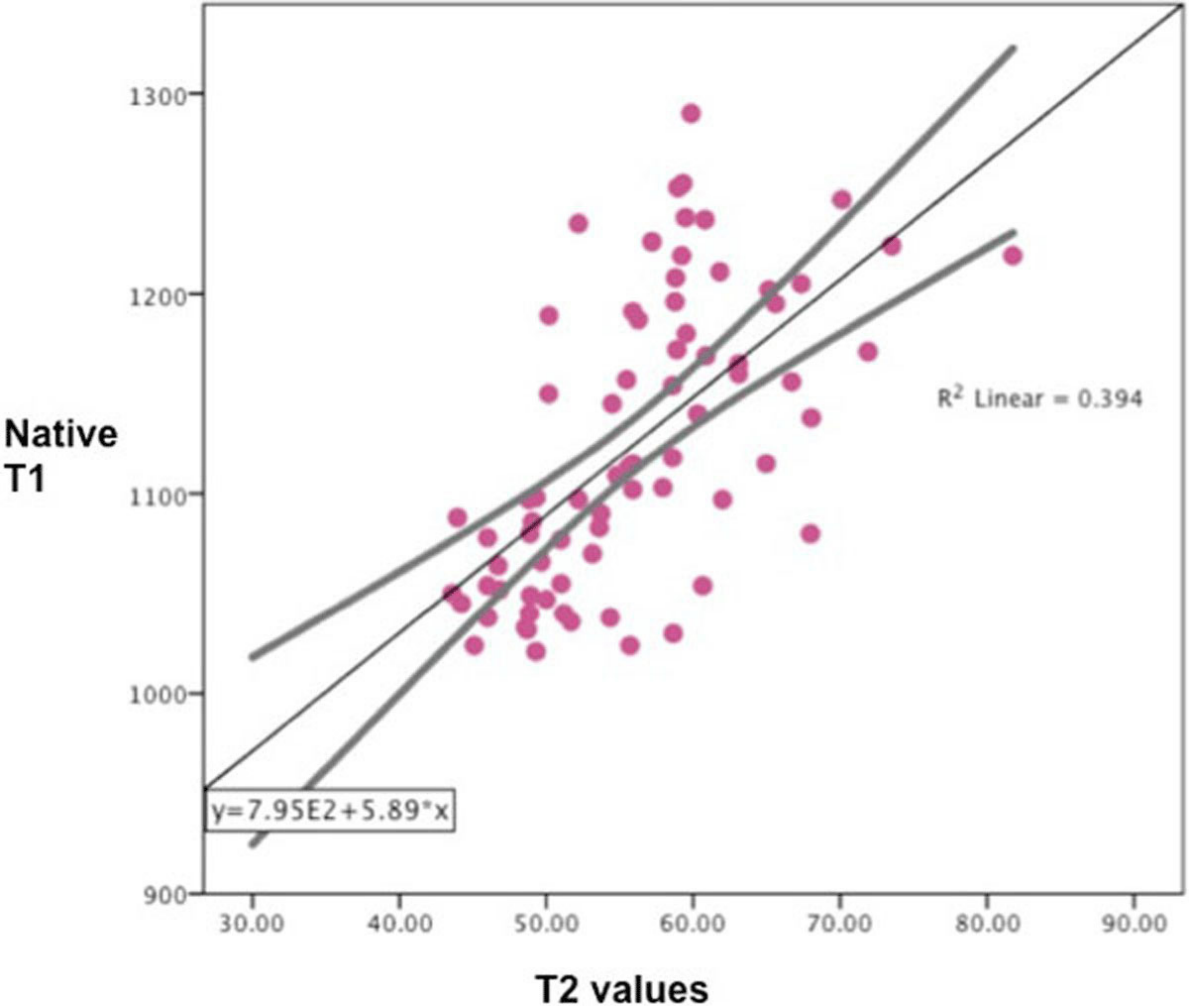
**There was a linear relationship between native T1 and average T2**.

Patients showed impaired global LV systolic function compared to controls (control vs. SLE vs. RA vs. SS vs. WG, % = 62 ± 5 vs. 54 ± 12 vs. 60 ± 8 vs. 53 ± 8 vs. 56, p = 0.02). There were no differences in LV volumes and LV mass between controls and patients' group. Patients showed LGE (SLE: n = 19 (41%); RA: n = 2 (12%); SS:n = 5(56%); WG: n = 4 (57%), which was of non-ischaemic pattern in presentation. Oedema ratio was increased in all systemic inflammatory conditions (p < 0.0001). Native T1 values were significantly raised in patients compared to controls (controls vs. SLE vs. RA vs. SS Vs. WG, native T1 (msec): 1048 ± 26 vs. 1170 ± 52 vs. 1118 ± 52 vs. 1199 ± 81 vs. 1151 ± 57, p < 0.0001). There were no significant regional variations in T2 values between the segments of the mSAX slice in controls with concordant SD between the segments. Patients showed raised native T2 values (native T2 (msec, average of all segments per mSAX): 48 ± 3 vs. 58 ± 7 vs. 54 ± 5 vs. 53 ± 3 vs. 57 ± 8, p < 0.001). Patients also showed regional differences in T2 values (range of native T2 values within mSAX, maximum vs. minimum (msec): SLE: 61 ± 6 vs 54 ± 8; RA: 58 ± 6 vs. 49 ± 3, SS: 59 ± 3 vs 51 ± 3, p < 0.01). There was a linear relationship between native T1 and average T2 (r = 0.63, p < 0.001) (Figure 1). Quantitative native T1 and T2 values were concordant with oedema ratio (r = 0.56 and r = 0.52 respectively, p < 0.001) and the presence of LGE (r = 0.28 and r = 0.34 respectively, p < 0.01).

## Conclusions

We demonstrate that native T1 and T2 values are increased in patients with systemic inflammation compared to controls. We also demonstrate that T2 values have regional differences in the presence of diffuse involvement. Native T1 and T2 values may serve as an early marker of myocardial injury due to diffuse fibrosis and low grade of oedema.

## Funding

We would like to acknowledge Department of Health via the National Institute for Health Research (NIHR) comprehensive Biomedical Research Centre award to Guy's & St Thomas' NHS Foundation Trust in partnership with King's College London and King's College Hospital National Health Service Foundation Trust. Dr. Rocio Hinojar was supported by the Fundacion Alfonso Martin Escudero.

